# Photochemical Reduction of Silver Precursor and Elastomer Composite for Flexible and Conductive Patterning

**DOI:** 10.3390/ma12233809

**Published:** 2019-11-20

**Authors:** Seok Young Ji, Hoon-Young Kim, Sung-Hak Cho, Won Seok Chang

**Affiliations:** 1 Department of Nano Mechanics, Nanomechanical Systems Research Division, Korea Institute of Machinery and Materials, 156 Gajeongbuk-Ro, Yuseong-Gu, Daejeon 34103, Korea; ji10047@kimm.re.kr; 2 Department of Nano-Mechatronics, Korea University of Science and Technology (UST), 217 Gajeong-Ro, Yuseong-Gu, Daejeon 34113, Korea; shcho@kimm.re.kr; 3 Department of Laser & Electron Beam Application, KIMM, Korea Institute of Machinery and Material, 156 Gajeongbuk-Ro, Yuseong-Gu, Daejeon 34103, Korea; hykim@kimm.re.kr

**Keywords:** laser reduction, heat-sensitive substrate, Ag precursor, poly(styrene-block-butadiene-block-styrene) (SBS)

## Abstract

The development of ink-based printing techniques has enabled the fabrication of electric circuits on flexible substrates. Previous studies have shown that the process method which uses a silver (Ag) precursor (AgCF_3_COO) and electrospun poly(styrene-block-butadiene-block-styrene) (SBS) can yield patterns with high conductivity and stretchability. However, the only method to reduce the Ag precursor absorbed in SBS is chemical reduction using a toxic solution. Here, we developed a process to fabricate a high-conductivity pattern via laser reduction by photo-chemical reaction without toxic solutions. The Ag precursor was absorbed in electrospun SBS to form a composite layer (composite SBS) with modified properties, that could more effectively absorb the photon energy than SBS without the Ag precursor. We analyzed the properties of this material, such as its light absorption coefficient, heat conductivity, and the density of both SBS and composite SBS to allow comparison of the two materials by numerical simulation. In addition, we fabricated patterns on highly heat-sensitive substrates such as burning paper and a polyethylene terephthalate (PET) thin film, as the pattern can be implemented using very low laser energy. We expect the proposed approach to become a key technology for implementing user-designed circuits for wearable sensors and devices on various flexible substrates.

## 1. Introduction

In recent years, photolithography-free electrode fabrication methods have attracted considerable attention for potential use as components in wearable devices for humans, such as electrodes [1], sensors [2], displays [3], organic field-effect transistors [4], and flexible electronics [5]. Printing technologies, such as inkjet printing [6], screen printing [7], flexography [8], and gravure [9] approaches, have made it feasible to manufacture conductive electrodes in large areas at a moderate cost. Inkjet printing [10] and screen printing [7] are popular deposition methods for photolithography-free processes and enable electrodes to be fabricated on a flexible substrate; however, these methods have limitations with respect to high-resolution electrode patterning. Laser direct writing (LDW) [11,12,13,14] with metal nanoparticle ink is a promising alternative. LDW methods are implemented by subjecting metal nanoparticle ink to laser sintering [15] and the metal-oxide nanoparticle ink to laser reduction [16]. LDW with nanoparticle ink can effectively be used to perform direct patterning on various substrates with high-resolution linewidth. Most target materials for electrodes have been noble metals such as Ag and Au owing to the high oxidation potential energy of Ag (0.799 V) and Au (1.52 V) ions. Copper (Cu), nickel (Ni), and aluminum (Al), which are inexpensive metals, are easily oxidized in air because of their low oxidation potential energy. Several researchers have fabricated Cu and Ni metal electrodes as alternatives to noble metals, by reducing copper and nickel oxide nanoparticles using a pulsed laser [17] or intense pulsed light [18]. However, a high-energy laser is necessary to either sinter or reduce metal or metal-oxide nanoparticle ink, such that the substrate is thermally influenced by the laser. Hence, substrates with thermal durability have been restrictively applied to fabricate flexible devices. Existing commercially available thin substrates such as polyethylene terephthalate (PET), polypropylene (PP), and textile exhibit weak characteristics for heat even in conventional printing. 

SBS is a three-dimensional stretchable fiber, which, after absorbing the Ag precursor, can be used to fabricate a pattern with various conductivities in the three-dimensional form through reduction [19,20,21]. Song et al. have fabricated selective patterns on SBS using a nozzle printer [19]. However, nozzle printing cannot implement patterns below the nozzle size. The main disadvantage is that hydrazine, the solution used to reduce the Ag precursor chemically, is a very toxic solution. We explored the reduction of the Ag precursor absorbed in SBS using a laser, and without this toxic solution.

In this study, we fabricated conductive patterns via the laser reduction of an Ag precursor on composite SBS comprising a mixture of SBS and the Ag precursor. The Ag precursor was easily absorbed into the SBS and is more affordable than metal nanoparticles. Moreover, absorption of the Ag precursor caused the characteristics of SBS to change such that it became possible to use a low-power laser beam to easily induce a photochemical reaction. Here, the term “reductive sintering” refers to the phenomenon wherein laser reduction occurs by photochemical reaction immediately before sintering via thermal reaction. We verified that the reduction of Ag ions in composite SBS occurred efficiently by photochemical reaction and reduced Ag particles sintered via a thermal reaction using a laser in the presence of air. This method enables high-resolution Ag patterns to be generated from composite SBS thin films by a vacuum-free, lithography-free, and solution-processable route.

## 2. Materials and Methods 

### 2.1. Experimental Setup

A continuous wave (CW) laser (mpc6000, Ventus, Cheshire, England) was used for the selective laser reduction of composite SBS. It has wavelength of 532 nm, a maximum power of 1.6 W, beam diameter of 1.5 mm ± 0.1 mm, power stability of <0.4% RMS, and M^2^ of <1.1. A galvano scanner (intelliSCAN^®^10, SCANLAB, Puchheim, Germany), which has a scanning speed of 1.0 mm s^−1^ – 3.0 m s^−1^, scan angle of ±22°, scan area of 50 × 50 mm^2^, and nonlinearity of <3.5 mrad, was used.

### 2.2. Numerical Analysis and Measurement of Thermal Properties

The finite element method (FEM), which uses the commercial package COMSOL 5.3a (COMSOL Multiphysics^®^, Burlington, VT, USA), was used to simulate the heat transfer properties of the material by numerical analysis. We measured the specific heat of the materials using modulated differential scanning calorimetry (MDSC). MDSC thermograms were obtained using a DSC204 F1 Phoenix (Labcompare, South San Francisco, CA, USA) under an N2 gas flow rate of 30 mL min^−1^ and a scanning rate of 5 °C during heating. Laser flash apparatus (LFA 467, NETZSCH, Selb, Germany) was used to measure the thermal diffusivity of the materials at various temperatures from 25 °C to 200 °C at 25 °C intervals. A He gas pycnometer apparatus (AccuPyc II, Micromeritics, Norcross, GA, USA) was used to measure the density of the materials. Fourier transform infrared (FTIR) spectra were recorded using an FTIR spectrometer (Nicolet 6700, ThermoElectron Corporation, Waltham, MA, USA).

### 2.3. Materials and Sample Preparation

Poly(styrene-block-butadiene-block-styrene) (SBS, styre-ne 30 wt.%), silver trifluoroacetate (98%, AgTFA), chloroform (99.9%), and acetone were purchased from Sigma-Aldrich. All chemicals were used as received without further purification. SBS was dissolved in chloroform to obtain a 10 wt % solution and then deposited by spin-coating. The silver precursor (AgTFA : SBS = 1 : 0.03) was dissolved in acetone at a solid concentration of 1 g mL^−1^.

### 2.4. Patterning Process 

**Figure 1:**
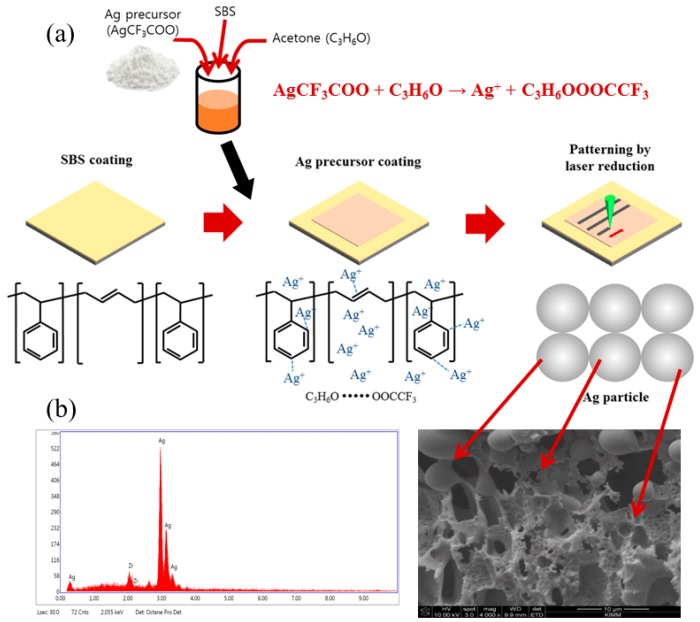
Schematic of the patterning process (**a**) and EDX analysis and SEM image of pattern obtained by laser reduction of Ag precursor (**b**).

Figure 1a depicts the flow and mechanism of the laser reduction patterning process in detail. First, SBS was spin-coated onto the substrate, followed by drying in an oven at 60 °C for 5 min. The thickness of the SBS films was 50 μm. SBS has a connatural bonding structure and the solvent was removed during spin coating and drying processing. Ag ions formed in the solution when the Ag precursor was dissolved in acetone as shown in the following Equation:

(1) $${\mathrm{AgCF}}_{3}\mathrm{COO}\;+\;C_{3}H_{6}O\;\to \;{\mathrm{Ag}}^{+}\;+\;C_{3}H_{6}OOOCCF_{3}$$

Trifluoroacetate anions (CF_3_COO^−^) undergo ion-dipole interaction with acetone (C_3_H_6_O), enabling rapid absorption of both, the Ag precursor and acetone, into the SBS [19,20]. When a coating of the Ag precursor solution is applied to the SBS by bar coating, the Ag ions contained in the solution either interact with the SBS bondings or remain absorbed in the SBS.

Fundamentally, SBS rubber can be directly dissolved using a solution of the Ag precursor in acetone even though the SBS is not completely soluble in pure acetone. However, the addition of a small amount of SBS (Ag precursor : SBS = 1 : 0.03) to the Ag precursor solution in acetone prevents the SBS from dissolving. Moreover, this solution is absorbed into the SBS and inhibits swelling phenomena [19]. Finally, selective laser irradiation of the SBS-absorbed Ag precursor removes the bonding structures of the SBS via a photochemical reaction, and the electrons generated by the removal of the bonding structures are transferred to the Ag ions, which are transformed into Ag nanoparticles. Sequentially, these Ag nanoparticles were sintered by the laser to change into Ag grains to form a conductive pattern. SEM was used to confirm the formation of Ag grains in the pattern (Figure 1b). Lastly, we conducted energy dispersive X-ray (EDX) analysis to verify that the grains observed by SEM are in fact silver.

## 3. Results and Discussion

**Figure 2:**
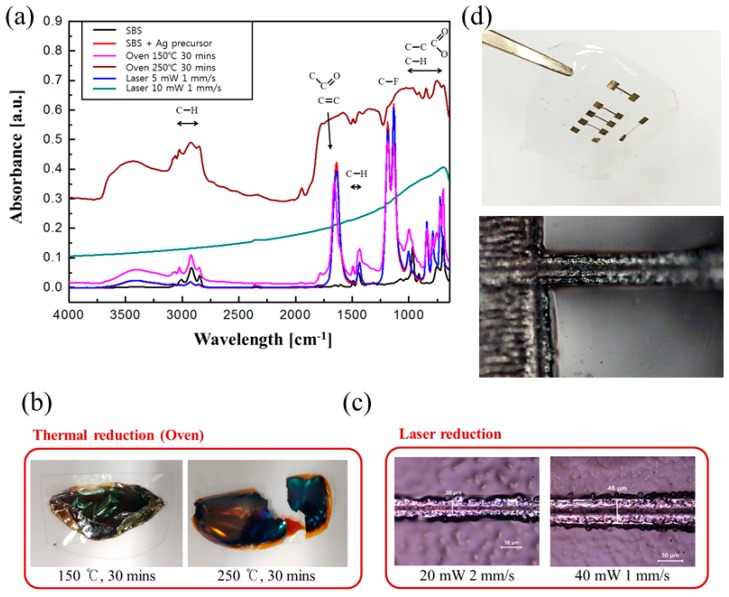
FTIR spectra of SBS (black), composite SBS (red), Ag precursor thermally reduced in an oven at 150 °C (pink) and 250 °C (brown) for 30 min, Ag precursor reduced using a laser at a laser power of 5 mW (blue) and 10 mW (bluish green) at a scan speed of 1 mm s^−1^ (**a**). Thermal heating of composite SBS in the oven at 150 °C and 250 °C for 30 min (**b**). Optical images of top view of the electrode line after laser irradiation on composite SBS (**c**). Fabricated electrodes with various line widths (30–400 μm) by laser reduction (**d**).

We performed FTIR analysis to confirm whether reduction of the Ag precursor in the SBS occurred by laser irradiation (Figure 2a). To allow the Ag ions to obtain electrons, it is necessary to generate electrons by breaking the bonding structure of SBS; that is, laser irradiation should have removed all SBS bonds. SBS has various bonding structures, as confirmed by the absorbance bands on the FTIR spectrum. Moreover, the absorbance corresponding to C−F stretching at 1128 cm^−1^ and 1182 cm^−1^ specifically confirms the presence of the Ag precursor inside the SBS [20]. The absorbance results obtained after laser irradiation of the composite SBS using 5 mW of laser power (laser scan speed of 1 mm s^−1^) is identical to the FTIR result for the composite SBS. In order for Ag ions to become an Ag particle, the generated electrons must be obtained by breaking the bond of the complex SBS by a photochemical reaction with the laser. We confirmed from FTIR that the photochemical reaction did not occur. However, when we irradiated the laser above a certain laser power (10 mW) such that the photochemical reaction occurred, the bonding structures in the SBS were broken by the photochemical reaction, and laser reduction occurred when the electrons were transferred to Ag ions. As a result, the FTIR spectra reveal that all the existing absorbance peaks of SBS disappear. Exposure of the Ag precursor to laser irradiation can cause either a photochemical or a thermal reaction. Provided that the reduction of the Ag precursor occurs by the thermal reaction when exposed to the laser, it would be possible to reduce the Ag precursor using a heating device such as a hot plate or oven. Therefore, we heated the composite SBS in the oven at 150 °C and 250 °C for 30 min, respectively. Heating the composite SBS above 250 °C in the oven caused the composite SBS to either dissolve or disintegrate. As a result, both composite SBS samples heated in the oven were blackened and were non-conductive (Figure 2b). As mentioned above, the FTIR spectra confirmed that the bonding structure of composite SBS cannot be broken by a thermal reaction. In addition, the spectra also confirmed that the Ag precursor is reduced by electrons generated when the bonding structure is broken. On the other hand, laser irradiation can fabricate the electrode using low laser power (Figure 2c). The results show that it is possible to fabricate fine conductive patterns on the electrospun SBS via laser reduction by photo-chemical reaction without toxic chemical reduction. Figure 2d shows the electrode patterns with various line widths (30–400 μm) after laser reduction on SBS. 

SBS can be easily coated on flexible substrates such as PET, PC, PI, and even on paper, and the pattern can be realized with very low laser power. In order to confirm that we can fabricate the patterns without thermal damage on substrate, we used COMSOL 5.3a to simulate the heat transfer process of the materials by laser before we conducted the experiment. FEM analysis for heat transfer by a laser source is defined by the following transient energy transport Equation:

(2) $$\rho C_{p}\frac{\partial T}{\partial t}+𝛻\times \left(-k𝛻T\right)=Q$$

The heat source term *Q* represents the heat generated by the laser absorbed in the material, and consists of the absorption coefficient (α), peak power of the laser (*P*), the reflectivity of the material (*R*), and its intensity distribution in the horizontal and depth directions. The intensity in both these directions has Gaussian distribution characteristics and can be expressed with the Beer–Lambert law, as in Equation (3):

(3) $$Q=\left(1-R\right)P\alpha \;\times \exp\left(-2\frac{x^{2}}{2\tau^{2}}\right)\times \exp\left(-\alpha z\right)$$

where *τ* represents the radius of the laser beam.

**Figure 3:**
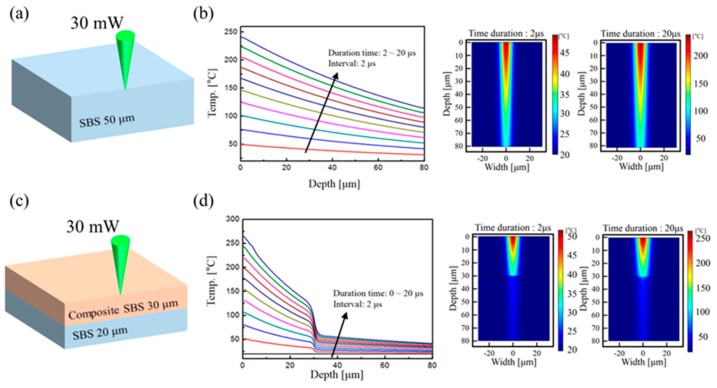
Schematics of simulation model of SBS (**a**) and composite SBS and SBS (**c**). Plot of the temperature change in depth (left) after laser irradiation from 0 μs to 20 μs (interval of 2 μs) and the temperature distribution in the vertical section of the substrate (right) after laser irradiation at 2 μs and 20 μs on the SBS (**b**) and composite SBS and SBS (**d**), respectively.

The dimensions of the materials in the samples and simulation results are presented in Figure 3. SBS was applied to the substrate by spin coating to a thickness of 50 μm. In addition, the Ag precursor was absorbed into SBS up to 30 μm thickness by bar coating. Thus, we simulated SBS only (Figure 3a–b) and composite SBS and SBS on the PET substrate (Figure 3c–d). The simulation used a laser beam diameter of 20 μm to irradiate the surface of the material, and two-dimensional modeling. As shown in Table 1, we measured the thermal and optical properties of SBS and composite SBS directly and applied it to the simulation.

Figure 3b,d shows a plot (left) of the temperature change in depth after laser irradiation from 0 μs to 20 μs (interval of 2 μs) and a sectional graphic (right) of the temperature distribution after laser irradiation at 2 μs and 20 μs of the SBS and composite SBS on SBS, respectively. In the case of SBS only, heat is transferred to the substrate. However, because the characteristics of composite SBS differ from those of SBS, exposure to the laser causes it to absorb almost all the heat, and no heat is transferred to the SBS. Consequently, the simulation results showed that heat was not transferred to the substrate by composite SBS. 

**Figure 4:**
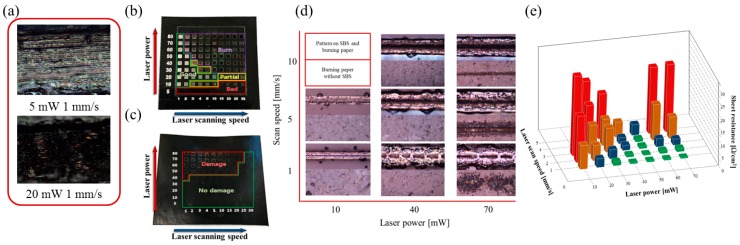
Optical microscopy (OM) image of burning paper after irradiating a laser with laser powers of 5 mW and 20 mW, scan speed of 1 mm s^−1^ (**a**); Patterns fabricated on the burning paper by varying the laser power from 5 mW to 80 mW and the scan speed from 1 mm s^−1^ to 30 mm s^−1^ (**b**) and burning paper with the composite SBS removed after pattern fabrication according to the laser power and scan speed on composite SBS (**c**); Optical image of top view of fabricated patterns (top image) and burning paper by laser reduction of the Ag precursor with various laser parameters (bottom image) after removal of the SBS composite (**d**); Sheet resistance graph of patterns fabricated by laser reduction with various laser parameters (**e**).

We subsequently proceeded with laser reduction on burning paper to verify that a conductive pattern could be fabricated on a heat-sensitive substrate without thermal damage. Figure 4a shows an optical microscopy (OM) image of the burning paper after laser irradiation at 5 mW and 20 mW of laser power. Assuming that the laser passes through the composite SBS and attacks the burning paper, we expected the burning paper to be damaged during pattern fabrication. Figure 4b shows the patterns fabricated on the burning paper by adjusting the laser power from 5 mW to 80 mW at a scan speed from 1 mm s^−1^ to 30 mm s^−1^. The patterns could not be fabricated using an irradiating laser of 5 mW or 10 mW above a scan speed of 15 mm s^−1^ because the laser power was insufficient to trigger the photochemical reaction. Increasing the laser power to 10 mW or above was sufficient to allow the photochemical reaction to occur and the pattern to be formed. However, the higher the laser power, the more intense was the burning phenomenon of composite SBS. In addition, an increase in the scan speed reduces the size and amount of Ag grains as the laser-induced thermal reaction of the Ag nanoparticles induced by the photochemical reaction becomes less prominent. Figure 4c shows the status of the burning paper with the composite SBS removed after fabricating the pattern. We verified whether the substrate was damaged by the laser. When the laser power is less than 30 mW, the laser is absorbed by the photochemical reaction and the laser sintering process, and the substrate remains thermally unaffected. Thermal damage is confirmed to have been induced when the laser power exceeds 40 mW at a scan speed of 1 mm s^−1^. 

However, when the scan speed exceeds 2.0 mm s^−1^, the accumulated amount of laser power is reduced, and the substrate is not damaged. Figure 4d shows an OM image of patterns for various laser parameters. The images in the top row show the pattern on the burning paper, and those in the bottom row show the burning paper from which the composite SBS was removed. At a laser power of 10 mW, the fabricated pattern width was small, and the substrate was not damaged. When the scan speed exceeded 10 mm s^−1^, the laser power was insufficient to transfer energy to the composite SBS due to the speed, and the photochemical reaction did not occur. The pattern had a burnt appearance due to a shortage of Ag grains caused by insufficient laser sintering by the thermal reaction. For a laser power exceeding 40 mW, the laser energy promoted the thermal reaction to generate a large amount of Ag grains during laser sintering; however, the burning paper was damaged by the laser. Figure 4e shows the sheet resistance graph to confirm the conductivity of the patterns fabricated by laser reduction with various laser parameters. For a sheet resistance of 100 Ω cm^−2^ or more, it was difficult to use the fabricated pattern as a conductive pattern, and the burning phenomenon mostly occurred (not shown on the graph). The sheet resistance decreased due to the growth of Ag grains by the thermal reaction when the laser power was increased, and the scan speed was decreased. However, if the thermal reaction was above a certain level, the substrate was damaged. These results confirm that it is possible to fabricate patterns on various substrates by adjusting the laser parameters for optimized line width and conductivity.

**Figure 5:**
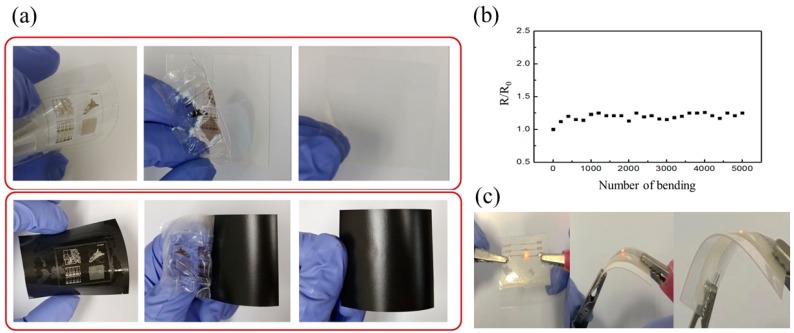
Various patterns fabricated on PET and burning paper and removal of the composite SBS (**a**); Measured resistance variation (R/R_0_) after the cyclic bending test (**b**); Bending test of the fabricated electric device after laser reduction of the Ag precursor (**c**).

Figure 5a shows images of various patterns fabricated on PET and burning paper. The line width of the patterns was 35 μm and 30 μm at laser powers of 50 mW and 30 mW and scan speeds of 5.0 mm s^−1^ and 2.0 mm s^−1^ on PET and burning paper, respectively. To confirm that the substrates were not damaged by the laser, we removed the composite SBS using a solvent. Next, cyclic tests were performed on an electrode printed on PET using a radius of curvature of 7 mm while monitoring the changes in the resistance. A pattern of area 20 mm × 0.5 mm was fabricated on the substrate, and copper tape was attached to the end of the two sides. The resultant change in resistance (Figure 5b) was minimal (<1.45%) throughout the 5,000-cycle test. Finally, we fabricated a micro-LED device via the laser reduction of the Ag precursor to verify that this process is suitable for flexible applications. As shown in Figure 5c, the micro-LED-mounted circuit was operated according to the input signal and showed that LED lighting was well-maintained during the bending state.

## 4. Conclusions

All processes, from materials synthesis to laser reduction, were performed under ambient conditions without photolithographic steps. We verified that Ag precursor and SBS composite is efficiently reduced by the photochemical reaction and sinters via a thermal reaction when exposed to laser irradiation. Therefore, we evaluated the optical and thermal properties of both SBS and composite SBS and simulated the thermal distribution that occurs when SBS and composite SBS are irradiated by the laser. Based on the simulation result that the thermal distribution by the laser is not transferred to the substrate, we fabricated various patterns on heat-sensitive substrates such as PET and burning paper. We then verified that the substrates were not damaged by the laser. A cyclic bending test and a bending test with a micro-LED confirmed that our process is applicable to flexible circuit fabrication. We expect the proposed approach to become a key technology for implementing user-designed flexible electronic devices in the near future.

## Figures and Tables

**Table 1:** Material properties of SBS and composite SBS.

Material		SBS	Composite SBS	-
Specific Heat	J (kg·K)^−1^	2871.1	2332.6	DSC 200 °C
Heat Conductivity	W (m·K)^−1^	0.162	0.270	Laser flash 200 °C
Density	g cm^−3^	0.9558	1.4523	-
Absorption Coefficient	m^−1^	4.5 × 10^6^	1.0 × 10^6^	Wavelength 532 nm
